# Cholera

**Published:** 1853-05

**Authors:** 


*Cholera.*—At last we are about to be rid of that scourge of the four quarters of the globe—the cholera; it has, we would fain believe, accomplished its work of death; fulfilled its mission, under Providence, and departed from among us. In no part of the United States does it prevail to any extent; it has spent its force—dried up the fountains of life in millions of subjects—swelled the bosom of the earth with its victims—made desolate the hearts and households of thousands of families, and gone forth to other climes—to other regions, to renew the contest for life or death with the sons and daughters of mortality. Cholera! its mere name sends a thrill of horror to the heart of the brave as well as the timid; to many it will convey sad recollections and heart-rending reminiscences, of friends, relatives and kindred, who were cut off from earth by the Great Destroyer. But why pursue the subject? Let the past be forgotten.

*Quis talia fando, * * temperet à lachrymis?*

** * * animus meminisse horret, luctuque refugit!*

We trust we have seen the last of this scourge; let us hope that it has departed from us forever!—*New York Med. Gazette.*

